# Characterization of rotary valve control vibration system for vibration stress relief applications

**DOI:** 10.1038/s41598-024-59970-z

**Published:** 2024-04-24

**Authors:** Guoqiang Zhou, Guochao Zhao, Hui Wang

**Affiliations:** 1 School of Mechanical Engineering, Liaoning Technical University, Fuxin, 123000 China; 2 Liaoning Provincial Key Laboratory of Large-Scale Industrial and Mining Equipment, Fuxin, 123000 China

**Keywords:** Rotary valve, Vibration system, Vibration characteristics, Parameters analysis, Experimental verification, Mechanical engineering, Electrical and electronic engineering

## Abstract

To enhance the vibration system characteristic distortion and pressure loss, we propose a novel rotary valve control vibration system. The paper presents the designed structural composition and generation mechanism of the rotary valve control vibration system. It also derives the mathematical model for the rotary valve distribution process and the overall system. The flow field inside the rotary valve is dynamically simulated using the multiple reference frame model, allowing for the determination of the change rule of the rotary valve’s output characteristics. An AMESim model was developed to analyze the vibration characteristics of the rotary valve control system. The effects of parameters such as inlet pressure, motor speed, and oil supply pump displacement were investigated. A rotary valve control vibration system experimental bench was constructed to experimentally verify the output characteristics of the rotary valve and the vibration characteristics of the system. The results indicate that the characteristic curve of the designed vibration system closely resembles a sinusoidal wave. Additionally, the rotary valve exhibits low pressure loss, making it more suitable for vibration stress relief applications. By appropriately increasing the inlet pressure and decreasing the motor speed, the vibration characteristics of the system can be improved.

## Introduction

Electro-hydraulic vibration excitation equipment through the control and actuating elements to generate usable vibration and liquid wave energy, with a large output power, significant flexibility and load adaptability and other advantages, not only to meet the vibration requirements of large loads, but also multi-dimensional vibration^1^. It is widely used in vehicles, machinery and aerospace^2,3^, rock crushing^4^, material vibratory conveying^5^ and other related engineering fields.

Electro-hydraulic excitation equipment is divided into valve-controlled vibration and valveless controlled vibration according to the way of excitation. Valve-controlled electro-hydraulic excitation system has a simple structure, high control accuracy, easy to realize automation and integration, and it is frequently used in engineering equipment and vibration research^6^. Valve-controlled electro-hydraulic excitation equipment have three basic structures^7^, which are classified as 2D-valve controlled^8^, slide-valve controlled^9,10^ and rotary-valve controlled^11,12^. The 2D valve controlled electro-hydraulic excitation apparatus vibrates by controlling the flow of liquid^13^. The slide valve changes the axial position of the valve core to regulate the flow rate, thus realizing vibration^14^. Electrohydraulic excitation equipment that is controlled by rotary valves generate vibrations through the alternating flow of the relative rotation of the valve core and valve sleeve^15,16^. Slide-valve controlled electrohydraulic excitation equipment require an expensive control valve and a complex internal flow that is prone to contamination and blockage^17^. In contrast, rotary valve-controlled electro-hydraulic excitation equipment has the advantages of adjustable amplitude, wider vibration frequency and high flow recognition rate^18,19^.

Therefore, specialists and academics have conducted relevant research on rotary valve and rotary valve control electro-hydraulic excitation systems. Yuan et al.^20^ investigated the switching characteristics of a high-speed rotary valve and verified them by computational fluid dynamics model and experiments. Agh et al.^21^ design improvements to the opening shapes of a rotary proportional flow control valve improved the fuel metering accuracy and the ultimate control accuracy of the system. Xu et al.^22^ proposed a novel high-frequency two-dimensional rotary valve in which the fluid is discretized in the form of fluid pulse width modulation to achieve flow distribution according to the demand of the system actuator. The flow field model with different valve port angles was established, and the throttling loss of the valve port was obtained and verified by simulation and experiment. Zhu et al.^23^ investigated the complete opening and closing process of a direct-drive rotary control valve with a novel structure by using a sliding mesh model and a moving region grid method, and analyzed the flow characteristics and fluid torque of the valve. Liu et al.^24^ leakage characteristics of a fully-rotary valve energy recovery device (FRV-ERD) which is a novel of isobaric ERD are studied numerically. Leakage variation of the fully-rotary valve (FRV) that is the key part of the device caused by pressure difference and geometric parameters is investigated. Ren et al.^25^ enhanced the 2D rotary valve in the horizontal type electro-hydraulic excitation system, analyzed the output characteristics, derived the analytical expressions for the operating frequency and excitation waveform, and proved that analytical expressions were consistent with the experimental data. Liu et al.^26^ proposed a novel wave generation device for electro-hydraulic exciter using rotary valve control and established a mathematical model of the wave generation device. Through experiments, the relationship between different parameters and the wave peak value was obtained, which broadened the application field of rotary valve-controlled electro-hydraulic exciters. Wang et al.^27–29^ designed electro-hydraulic exciter consisting of four-way rotary valve and miniature double-acting hydraulic cylinder, described the theoretical formulas for the flow area of various valve port shapes, simulated and analyzed the output vibration characteristics of the electro-hydraulic exciter, and experimentally verified the results. Ren et al.^30^ proposed a hydraulic vibration excitation system controlled by a novel of shock rotary vibrator, established a system model considering pipeline effect and compared it with the model without considering pipeline effect, and experimentally verified the validity of the proposed model. Liu et al.^31,32^ summarized the research progress of wave generating devices with wave structural impact experiment, pointed out the technical bottlenecks of existing wave generating devices in amplitude preservation and waveform control, and designed push plate type wave generating device based on rotary valve control, realizing a new utilization of electro-hydraulic wave excitation energy. The above plays a guiding role in the research of rotary valve control vibration system, but most of the researches focus on mathematical theory and model analysis, with only a few scholars’ researches focus on the vibration characteristics of rotary valve control vibration system.

In order to address the issues of vibration system output characteristic distortion and pressure loss, this paper presents a novel design of a rotary valve control vibration system that is better suited for vibration stress relief applications. The structural composition of the rotary valve control vibration system is designed, and the mathematical models for the rotary valve flow distribution process and the overall system are derived. The dynamic simulation of the internal flow field of rotary valve is carried out by using the method of multiple reference systems, and the change rule of the output characteristics of rotary valve is obtained. The AMESim model of rotary valve control vibration system is established, and the influence of multi-parameters on vibration characteristics is analyzed. Finally, the test bench prototype is built to experimentally verify the output characteristics of the rotary valve and the vibration characteristics of the system. The research results can lay the theoretical foundation and data support for application of vibration stress relief.

## Rotary valve control vibration system design and vibration mechanism

### Rotary valve design and principle

**Figure. 1:**
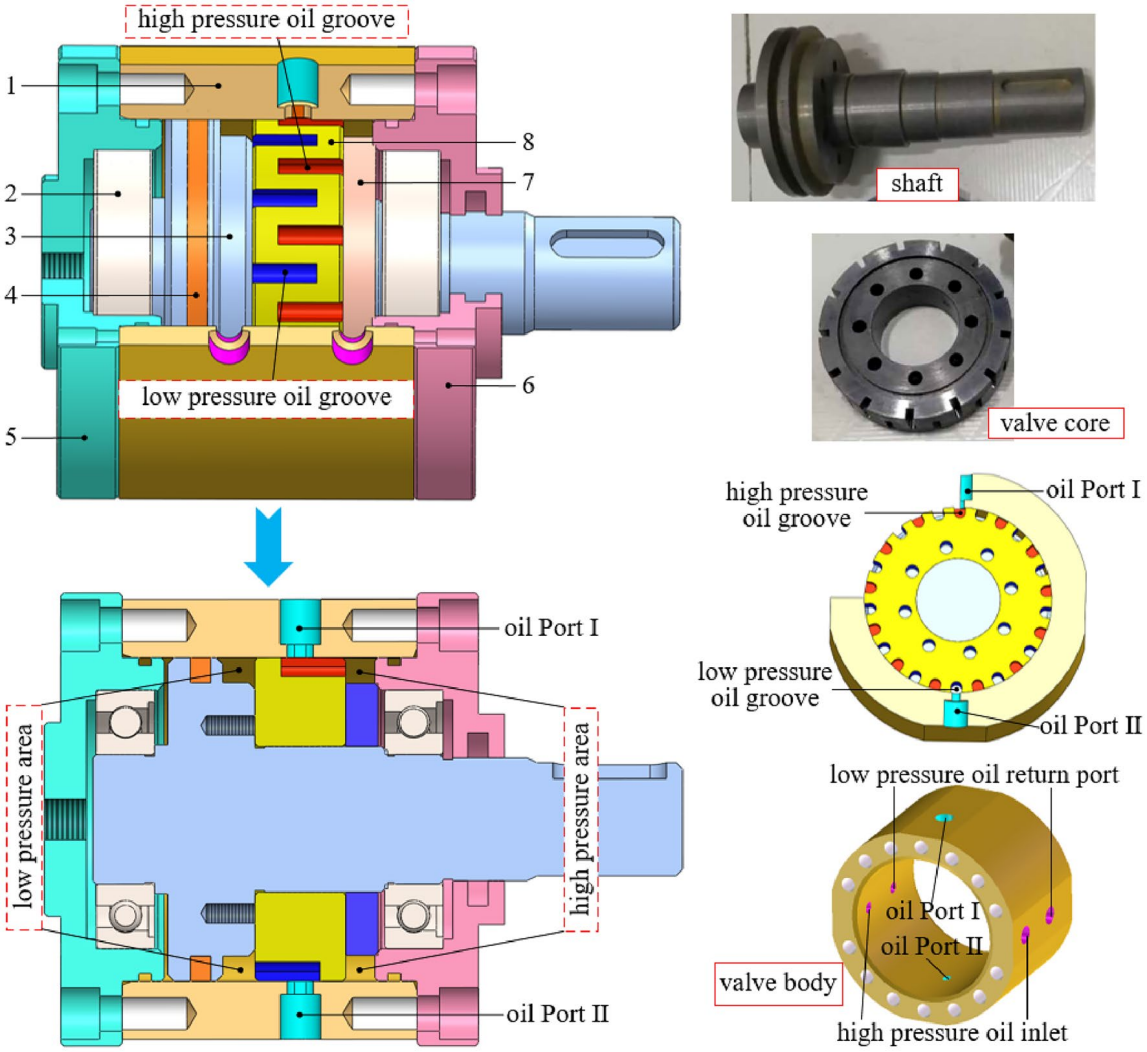
Rotary valve structural. 1. valve body; 2. bearings; 3. shaft; 4. glyd-ring; 5. back closure; 6. front closure; 7. gasket; 8. valve core.

The main components of the proposed rotary valve are the valve body, shaft, valve core and glyd-ring, and its structural principle is shown in Fig. 1.

From Fig. 1: the rotary valve valve core has 26 alternating oil grooves, 13 grooves on the left side are positioned in the low-pressure area of the external tank, and 13 grooves on the right side are positioned in the high-pressure area of the external oil supply pump. Four oil ports are arranged symmetrically on both sides of the valve body, and two oil ports are connected to external oil supply pumps to complete the input of high-pressure oil to the inside of the rotary valve. The other two ports are connected to the external tank to realize the low-pressure oil returning to the tank through the inside of the rotary valve. Two oil ports are symmetrically distributed at the upper and lower ends of the valve body, and the two ports are alternately connected to the high-pressure and low-pressure cavities of the excitation hydraulic cylinder. With the continuous rotation of the shaft, the drive valve core oil groove is alternately connected to the two oil ports at the upper and lower ends of the valve body. The valve core rotates 4 groove angles, the rotary valve completes an oil supply and return, the oil supply and return processes are independent of each other, do not affect each other and are carried out at the same time.

### Rotary valve control vibration system vibration mechanism

**Figure 2:**
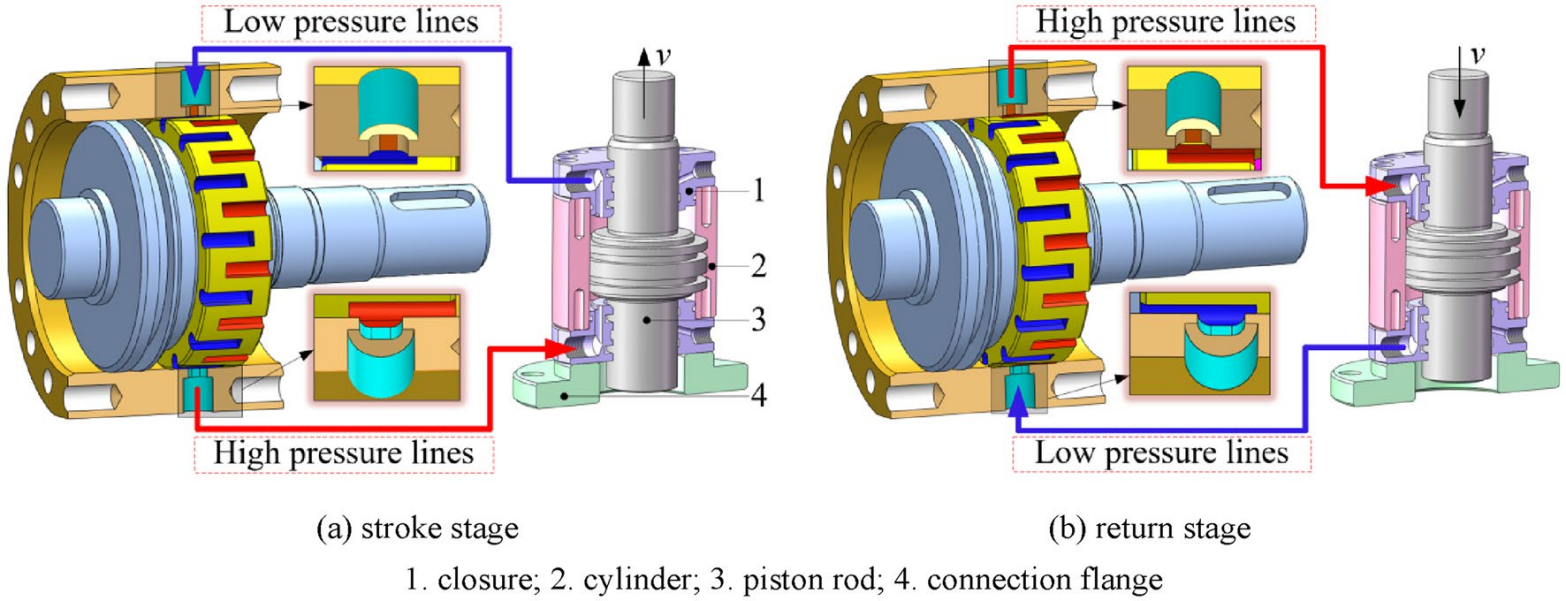
Rotary valve control vibration system.

The working process of the rotary valve control vibration system can be divided into two stages as shown in Fig. 2: the stroke stage when the piston rod of the vibration hydraulic cylinder rises (Fig. 2a) and the return stage when the piston rod of the vibration hydraulic cylinder falls (Fig. 2b).

Rotary valve control vibration system vibration mechanism: valve core high-pressure oil groove and the valve body at the lower end of the oil through the flow of high-pressure oil through the pipeline of the lower cavity of the hydraulic cylinder, resulting in the role of the hydraulic cylinder piston upward force is greater than the downward force, thereby promoting the piston rod up. Due to the special structure of the rotary valve, the low-pressure return groove of the valve core and the oil port at the upper end of the valve body are in communication, and the oil in the upper chamber of the hydraulic cylinder returns to the oil tank through the low-pressure oil groove. The above two processes are carried out simultaneously to complete the stroke phase of the hydraulic cylinder. As the motor drives the rotary valve to rotate, the through-flow relationship in the stroke stage is switched, the oil return in the stroke stage becomes the oil supply in the return stage, and the oil supply in the stroke stage becomes the oil return in the return stage, and the work of the stroke and the return stage is carried out alternately, so that the piston rod of the hydraulic cylinder produces continuous vibration.

## Mathematical modeling of rotary valve control vibration system

### Theoretical basis of rotary valve flow field analysis

The MRF model divides the fluid domain is split into stationary and rotating domains, and the junction is connected by an interface, which belongs to the rotating domains as well as the stationary domain, and the data between different regions are transferred through the interface. The following equations should be satisfied when performing MRF flow field analysis:

1. For an incompressible working medium, the mass conservation equation is:
  1 $$\frac{\partial (\rho u)}{{\partial x}} + \frac{\partial (\rho v)}{{\partial y}} + \frac{\partial (\rho w)}{{\partial z}}{ = 0}$$
  where *ρ* is the fluid density (kg/m^3^), *u*, *v*, and *w* are the components of fluid velocity in the *x*, *y*, and *z* directions, m/s.
2. The energy conservation equation is:
  2 $$\frac{{\partial \left( {\rho \phi } \right)}}{\partial t}{ + }\frac{{\partial \left( {\rho u\phi } \right)}}{\partial x} + \frac{{\partial \left( {\rho v\phi } \right)}}{\partial y} + \frac{{\partial \left( {\rho w\phi } \right)}}{\partial z} = \frac{\partial }{\partial x}\left( {\Gamma \frac{\partial \phi }{{\partial x}}} \right) + \frac{\partial }{\partial y}\left( {\Gamma \frac{\partial \phi }{{\partial y}}} \right) + \frac{\partial }{\partial z}\left( {\Gamma \frac{\partial \phi }{{\partial z}}} \right) + S$$
  where *ϕ* is a generic variable, Γ is a generalized diffusivity, *S* is a generalized source term.
3. Mesh conservation equation is:
  3 $$\frac{{\text{d}}}{{{\text{d}}t}}\int_{V} \rho \phi {\text{d}}V{ = }\frac{{\left( {\rho \phi V} \right)^{n + 1} - \left( {\rho \phi V} \right)^{n} }}{\Delta t}$$
  where *V* is any cell mesh volume, *n* is the current computation time step.

The cell mesh volume *V*^*n*+1^ on the *n* + 1st computational time step can be expressed as:

4 $$V^{n + 1} { = }V^{n} + \frac{{{\text{d}}V}}{{{\text{d}}t}}\Delta t$$

Let d*V*/d*t* = 0, then the mesh conservation equation can be rewritten as:

5 $$\frac{{\text{d}}}{{{\text{d}}t}}\int_{V} \rho \phi {\text{d}}V{ = }\frac{{V\left[ {\left( {\rho \phi } \right)^{n + 1} - \left( {\rho \phi } \right)^{n} } \right]}}{\Delta t}$$

### Mathematical modeling of rotary valve flow distribution process

According to the structure of the rotary valve, the center angle of a single oil groove *α* = π/2Z (Z is the number of oil grooves on the same side of the valve core, Z = 13), and the center angle of the two adjacent oil grooves on the same side is 4*α*. A vibration cycle, the oil port I, II through-flow change state is shown in Table 1. When the rotary axis drives the valve core to rotate at an angular velocity *ω*, the valve core angular displacement *θ* = *ωt*.

**Table 1:** Oil port I, II through-flow change state.

	0 ≤ *θ* < *α*	*α* ≤ *θ* < 2*α*	2*α* ≤ *θ* < 3*α*	3*α* ≤ *θ* < 4*α*
Oil port I				
Oil port II				

With the alternation of oil supply and return, the through-flow form in the two states is exactly the same, and the through-flow area in the oil supply and return phases can be expressed as^33^:

6 $$A_{v1} = A_{v2} = \left\{ {\begin{array}{*{20}l} {x_{v} R\theta } & {0 < \theta \le \alpha } \\ {x_{v} R\left( {2\alpha - \theta } \right)} & {\alpha < \theta \le 2\alpha } \\ {x_{v} R\left( {\theta - 2\alpha } \right)} & {2\alpha < \theta \le 3\alpha } \\ {x_{v} R(4\alpha - \theta )} & {{3}\alpha < \theta \le 4\alpha } \\ \end{array} } \right.$$

where *A*_*v*1_ is the area of oil supply stage flux (m^2^), *A*_*v*2_ is the area of oil return stage flux (m^2^), *x*_*v*_ is the length of the valve body oil port (m), *R* is the valve core radius (m).

According to the flow distribution principle of the rotary valve, the flow rate changes of oil port I and II in 1 vibration cycle are^34^:

7 $$\left\{ \begin{aligned} &Q_{{\text{a}}} = C_{{\text{d}}} x_{v} R\theta \cdot \sqrt {\frac{{2\left( {P_{{\text{s}}} - P_{{\text{a}}} } \right)}}{\rho }} \\ &Q_{{\text{b}}} = C_{{\text{d}}} x_{v} R\theta \cdot \sqrt {\frac{{2P_{{\text{b}}} }}{\rho }} \\ \end{aligned} \right.\quad t \in \left[ {0,\frac{{\text{T}}}{4Z}} \right]$$

8 $$\left\{ \begin{aligned} &Q_{{\text{a}}} = C_{{\text{d}}} x_{v} R\left( {2\alpha - \theta } \right) \cdot \sqrt {\frac{{2\left( {P_{{\text{s}}} - P_{{\text{a}}} } \right)}}{\rho }} \\ &Q_{{\text{b}}} = C_{{\text{d}}} x_{v} R\left( {2\alpha - \theta } \right) \cdot \sqrt {\frac{{2P_{{\text{b}}} }}{\rho }} \\ \end{aligned} \right.\quad t \in \left[ {\frac{{\text{T}}}{4Z},\frac{{\text{T}}}{2Z}} \right]$$

9 $$\left\{ \begin{aligned} &Q_{{\text{a}}} = C_{{\text{d}}} x_{v} R\left( {\theta - 2\alpha } \right) \cdot \sqrt {\frac{{2P_{{\text{a}}} }}{\rho }} \\ &Q_{{\text{b}}} = C_{{\text{d}}} x_{v} R\left( {\theta - 2\alpha } \right) \cdot \sqrt {\frac{{2(P_{{\text{s}}} - P_{{\text{b}}} )}}{\rho }} \\ \end{aligned} \right.\quad t \in \left[ {\frac{{\text{T}}}{2Z},\frac{{3{\text{T}}}}{4Z}} \right]$$

10 $$\left\{ \begin{aligned} &Q_{{\text{a}}} = C_{{\text{d}}} x_{v} R(4\alpha - \theta ) \cdot \sqrt {\frac{{2P_{{\text{a}}} }}{\rho }} \\ &Q_{{\text{b}}} = C_{{\text{d}}} x_{v} R(4\alpha - \theta ) \cdot \sqrt {\frac{{2\left( {P_{{\text{s}}} - P_{{\text{b}}} } \right)}}{\rho }} \\ \end{aligned} \right.\quad t \in \left[ {\frac{{3{\text{T}}}}{4Z},\frac{{\text{T}}}{Z}} \right]$$

where *Q*_a_ is the flow rate of oil port I (L/min), *Q*_b_ is the flow rate of oil port II (L/min), *C*_d_ is the flow coefficient, *P*_s_ is the inlet pressure (MPa), *P*_a_ is the output pressure of oil port I (MPa), *P*_b_ is the output pressure of oil port II (MPa).

### Mathematical equations for vibration systems

Hydraulic cylinder for the double rod piston cylinder, the effective area of the piston in both cavities *A*_*p*_ the same, ignoring all kinds of energy loss and leakage problems. Assuming that the pressure is equal at all points in the working chamber of the hydraulic cylinder, and the flow state of the oil port I, II is the same and stable, we can establish the flow balance equation and the load force balance equation of the hydraulic cylinder in 1 vibration cycle:

11 $$\left\{ \begin{aligned}{}& C_{d} x_{v} R\theta \cdot \sqrt {\frac{{2\left( {P_{{\text{s}}} - P_{{\text{a}}} } \right)}}{\rho }} = A_{P} \frac{{{\text{d}}y}}{{{\text{d}}t}} + \frac{{V_{1} }}{{B_{e} }}\frac{{{\text{dP}}_{a} }}{{{\text{d}}t}} \\ &C_{d} x_{v} R\theta \cdot \sqrt {\frac{{2P_{{\text{b}}} }}{\rho }} = A_{P} \frac{{{\text{d}}y}}{{{\text{d}}t}} - \frac{{V_{2} }}{{B_{e} }}\frac{{{\text{dP}}_{{\text{b}}} }}{{{\text{d}}t}} \\& A_{p} \left( {P_{{\text{a}}} - P_{{\text{b}}} } \right) = m\frac{{{\text{d}}^{2} y}}{{{\text{d}}t}} + B_{p} \frac{{{\text{d}}y}}{{{\text{d}}t}} + F_{L} \\ \end{aligned} \right.\quad t \in \left[ {0,\frac{{\text{T}}}{4Z}} \right]$$

12 $$\left\{ \begin{aligned} &C_{d} x_{v} R\left( {2\alpha - \theta } \right) \cdot \sqrt {\frac{{2\left( {P_{{\text{s}}} - P_{{\text{a}}} } \right)}}{\rho }} = A_{P} \frac{{{\text{d}}y}}{{{\text{d}}t}} + \frac{{V_{1} }}{{B_{e} }}\frac{{{\text{d}}P_{a} }}{{{\text{d}}t}} \\& C_{d} x_{v} R\left( {2\alpha - \theta } \right) \cdot \sqrt {\frac{{2P_{{\text{b}}} }}{\rho }} = A_{P} \frac{{{\text{d}}y}}{{{\text{d}}t}} - \frac{{V_{2} }}{{B_{e} }}\frac{{{\text{d}}P_{{\text{b}}} }}{{{\text{d}}t}} \\& A_{p} \left( {P_{{\text{a}}} - P_{{\text{b}}} } \right) = m\frac{{{\text{d}}^{2} y}}{{{\text{d}}t}} + B_{p} \frac{{{\text{d}}y}}{{{\text{d}}t}} + F_{L} \\ \end{aligned} \right.\quad t \in \left[ {\frac{{\text{T}}}{4Z},\frac{{\text{T}}}{2Z}} \right]$$

13 $$\left\{ \begin{aligned} &C_{d} x_{v} R\left( {\theta - 2\alpha } \right) \cdot \sqrt {\frac{{2(P_{{\text{s}}} - P_{{\text{b}}} )}}{\rho }} = A_{P} \frac{{{\text{d}}y}}{{{\text{d}}t}} + \frac{{V_{2} }}{{B_{e} }}\frac{{{\text{d}}P_{{\text{b}}} }}{{{\text{d}}t}} \\& C_{d} x_{v} R\left( {\theta - 2\alpha } \right) \cdot \sqrt {\frac{{2P_{{\text{a}}} }}{\rho }} = A_{P} \frac{{{\text{d}}y}}{{{\text{d}}t}} - \frac{{V_{1} }}{{B_{e} }}\frac{{{\text{d}}P_{{\text{a}}} }}{{{\text{d}}t}} \\ &A_{p} \left( {P_{{\text{a}}} - P_{{\text{b}}} } \right) = m\frac{{{\text{d}}^{2} y}}{{{\text{d}}t}} + B_{p} \frac{{{\text{d}}y}}{{{\text{d}}t}} + F_{L} \\ \end{aligned} \right.\quad t \in \left[ {\frac{{\text{T}}}{2Z},\frac{{3{\text{T}}}}{4Z}} \right]$$

14 $$\left\{ \begin{aligned} &C_{d} x_{v} R(4\alpha - \theta ) \cdot \sqrt {\frac{{2\left( {P_{{\text{s}}} - P_{{\text{b}}} } \right)}}{\rho }} = A_{P} \frac{{{\text{d}}y}}{{{\text{d}}t}} + \frac{{V_{2} }}{{B_{e} }}\frac{{{\text{d}}P_{{\text{b}}} }}{{{\text{d}}t}} \\ &C_{d} x_{v} R(4\alpha - \theta ) \cdot \sqrt {\frac{{2P_{{\text{a}}} }}{\rho }} = A_{P} \frac{{{\text{d}}y}}{{{\text{d}}t}} - \frac{{V_{1} }}{{B_{e} }}\frac{{{\text{d}}P_{{\text{a}}} }}{{{\text{d}}t}} \\& A_{p} \left( {P_{{\text{a}}} - P_{{\text{b}}} } \right) = m\frac{{{\text{d}}^{2} y}}{{{\text{d}}t}} + B_{p} \frac{{{\text{d}}y}}{{{\text{d}}t}} + F_{L} \\ \end{aligned} \right.\quad t \in \left[ {\frac{{3{\text{T}}}}{4Z},\frac{{\text{T}}}{Z}} \right]$$

where *A*_*p*_ is the effective action area of the two-cavity piston (m^2^), *B*_*e*_ is the effective bulk modulus of elasticity (MPa), *B*_*p*_ is the viscous damping coefficient of the piston and the load, and *F*_*L*_ is the arbitrary load force (N).

Among them:

15 $$\left\{ \begin{aligned} &V_{1} = V_{01} + A_{{\text{p}}} y \\ &V_{2} = V_{02} - A_{{\text{p}}} y \\ &V_{01} { = }V_{02} \\ \end{aligned} \right.$$

where *V*_1_ is the volume of the piston chamber of the hydraulic cylinder connected to the oil port I (m^3^), *V*_2_ is the volume of the piston chamber of the hydraulic cylinder connected to the oil port II (m^3^), *V*_01_ is the initial volume of *V*_1_ (m^3^); *V*_02_ is the initial volume of *V*_2_ (m^3^).

## Rotary valve flow field characterization

### Simulation condition settings

**Figure 3:**
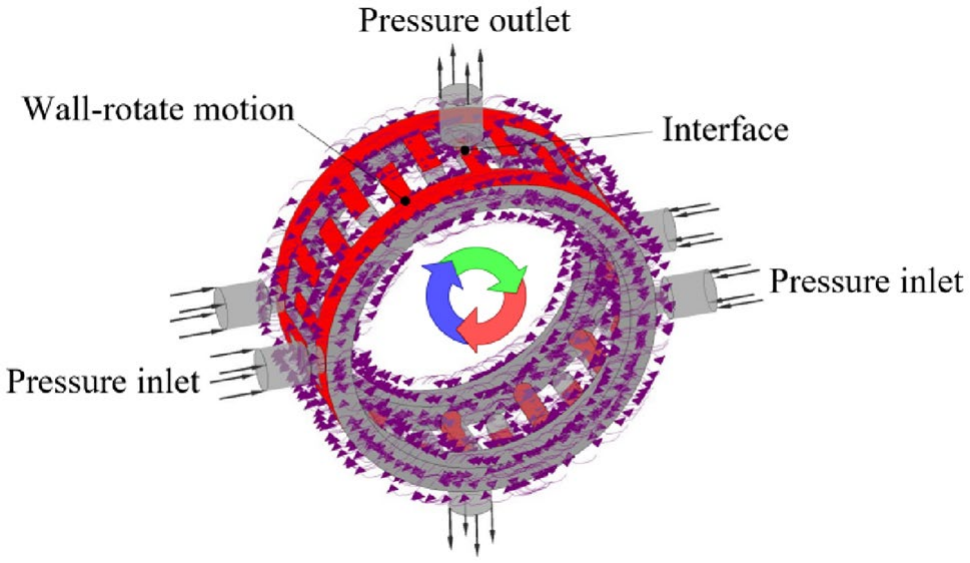
Condition settings.

The flow field inside the rotary valve is simulated dynamically using Fluent, and there is no mesh distortion in the rotating region, so it is feasible to simulate the flow field using MRF method. According to the valve core structure and functional symmetry, the three oil ports are set as the pressure boundary and the oil groove as the wall boundary of the slip domain, as shown in Fig. 3. The main simulation parameter settings are shown in Table 2.

**Table 2:** Main simulation parameters settings.

Project/unit	Numerical value
Time/s	0.5
Step size/s	0.0001
Oil density/kg m^−3^	890
Valve core speed/rpm	500
Inlet pressure/MPa	15
Outlet pressure/MPa	10

### Analysis of simulation results

**Figure 4:**
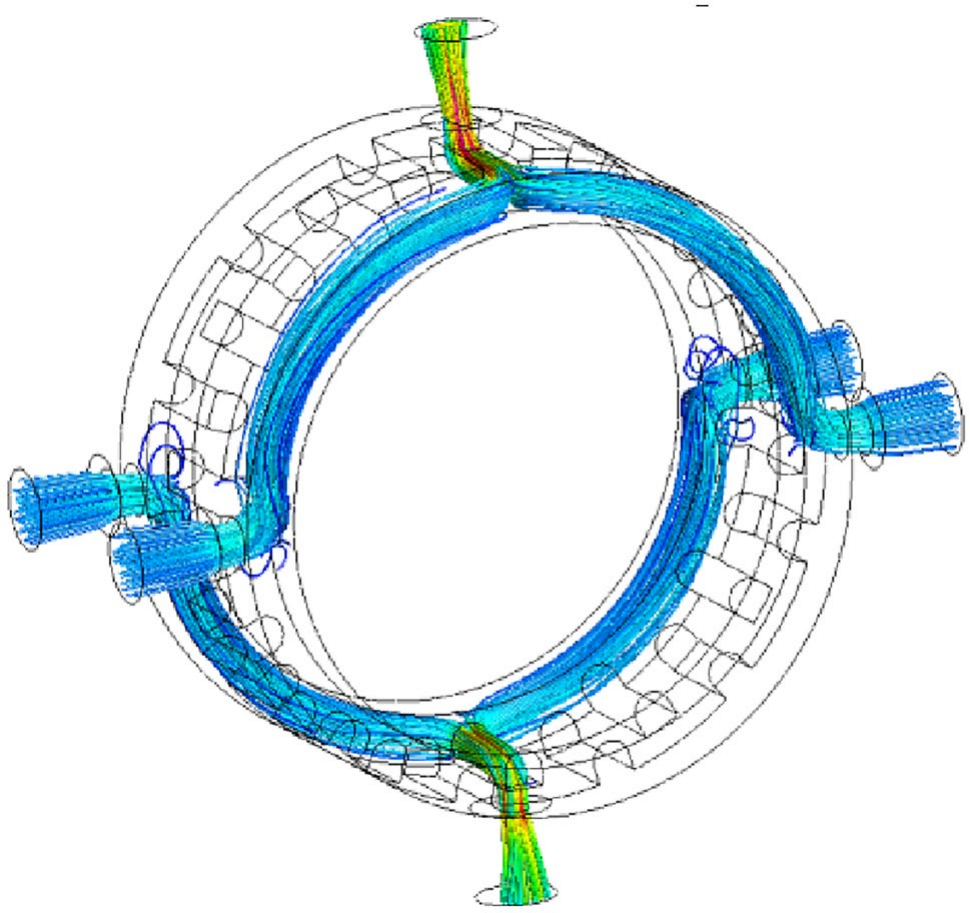
Flow velocity streamline diagram.

As can be seen from the streamline diagram in Fig. 4, the main area of interest in the flow field is centered on the connection between the valve core oil groove and the valve port.

**Figure 5:**
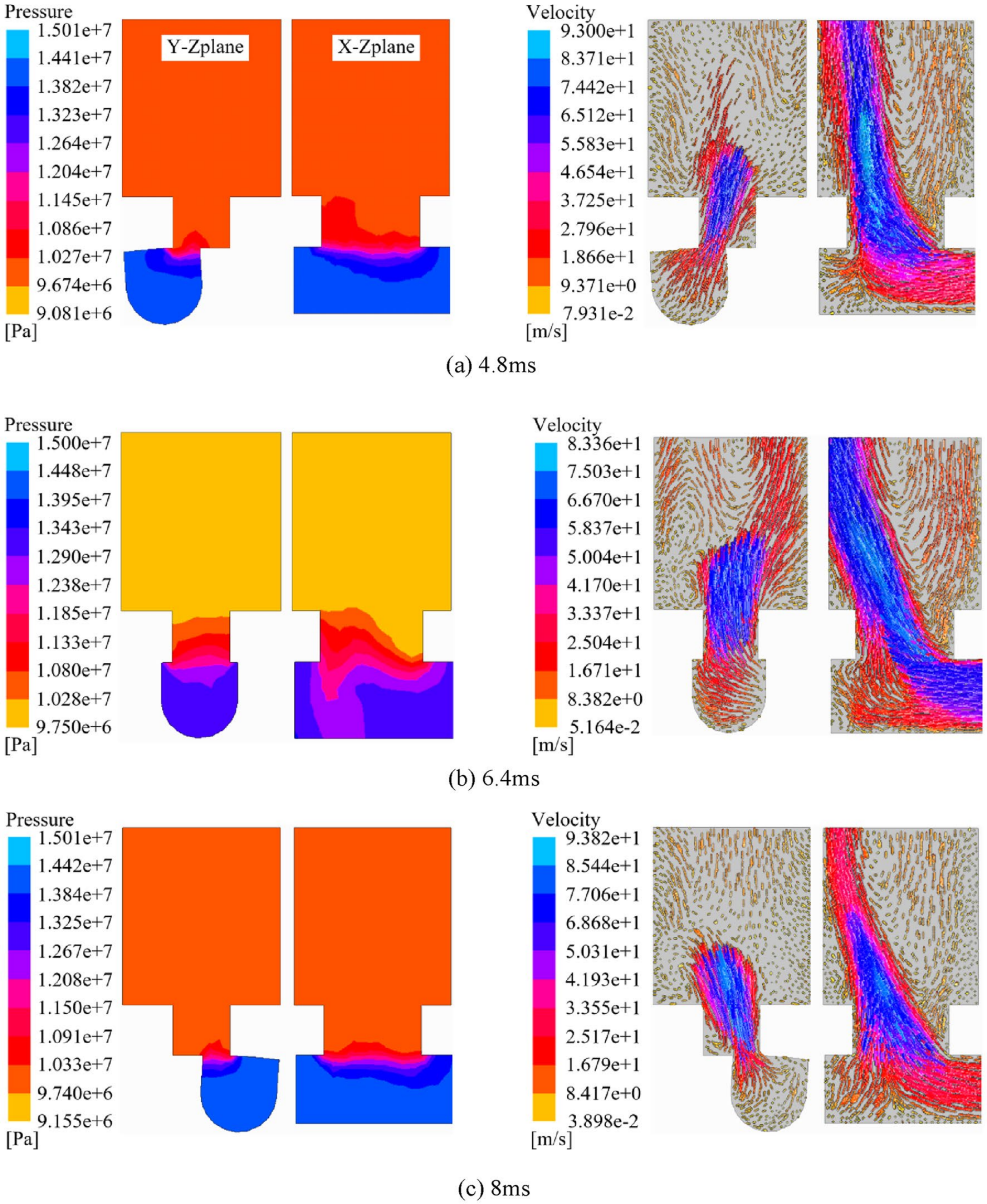
Pressure cloud plot and velocity vector plot of rotary valve.

The data in the high-pressure region is extracted in CFX-Post, and the Y–Z and X–Z planes are processed in cross-section to obtain the pressure cloud diagrams and flow velocity vector diagrams corresponding to three different moments, as shown in Fig. 5.

From Fig. 5a, it can be seen that the output pressure and flow rate of the rotary valve are 10.66 MPa and 90.65 m/s at 4.8 ms. From Fig. 5b, it can be seen that the valve opening is completely opened at 6.4 ms, and the output pressure and flow rate are 11.34 MPa and 79.72 m/s respectively. From Fig. 5c, it can be seen that the output pressure and flow rate are 10.74 MPa and 91.32 m/s at 8 ms respectively. It can be seen, the rotary valve of the first increase in the through-flow area after the decrease, the output pressure change rule and the through-flow area change rule is the same, the output flow rate change rule and the through-flow area change rule is opposite.

**Figure 6:**
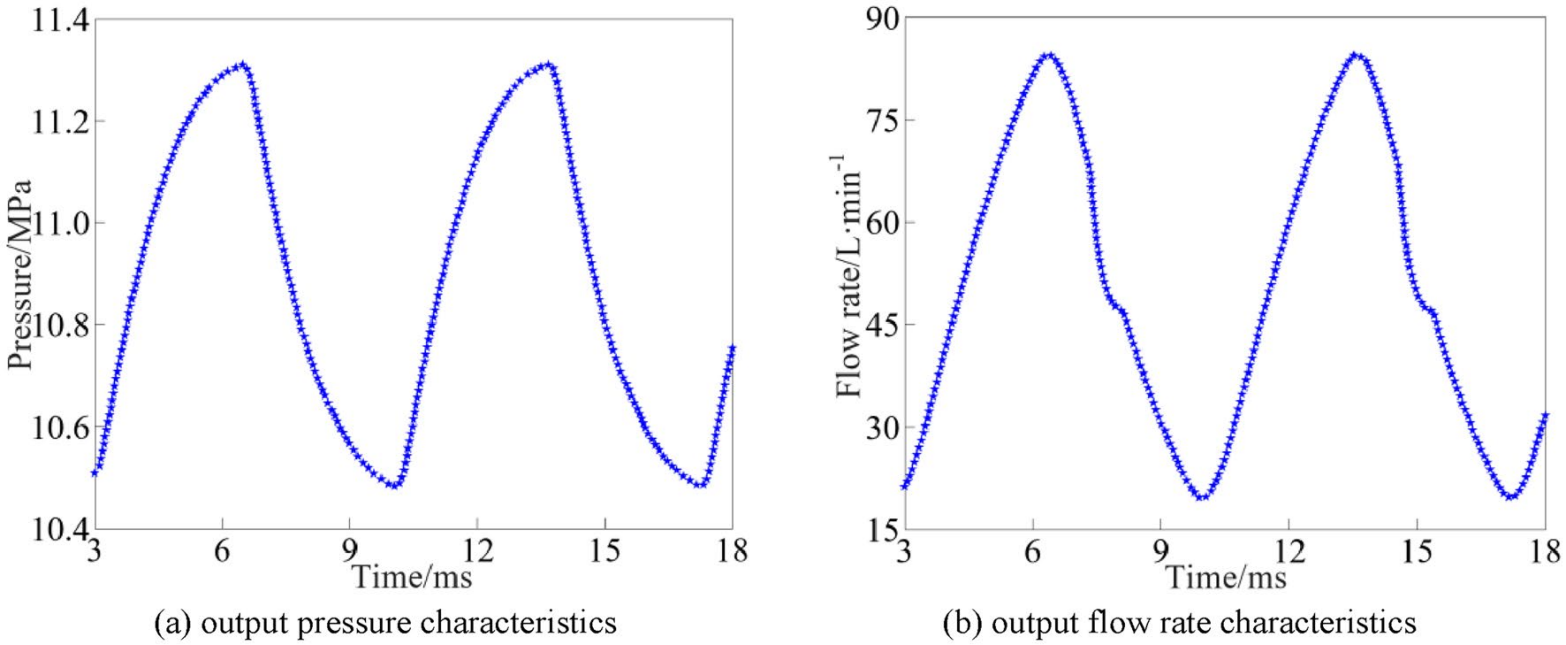
Rotary valve output characteristics curve.

The output pressure and flow rate simulation results of the rotary valve in the interval from 3 to 18 ms are selected and shown in Fig. 6.

As can be seen from Figs. 5 and 6, the rotary valve orifice undergoes the process of opening- fully open-closed-fully closed, with the through-flow area first increasing and then decreasing. When the rotary valve is fully open, the output pressure and flow rate reaches the peak, respectively, 11.34 MPa and 84.42L/min. The change rule of output pressure and flow rate is the same as the change rule of through-flow area.

## Characterization of rotary valve control vibration system

**Figure 7:**
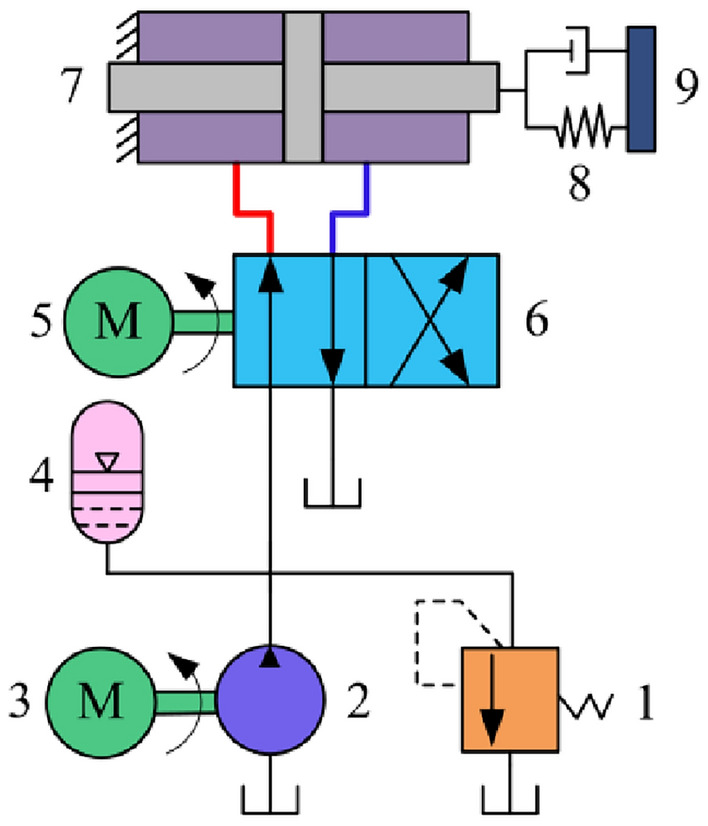
Schematic diagram of rotary valve controlled vibration system. 1. relief valve; 2, 3 oil supply pump; 4. accumulator; 5. servo motor; 6. rotary valve; 7. vibration hydraulic cylinder; 8, 9. load spring stiffness and damping.

The schematic diagram of rotary valve controlled vibration system is shown in Fig. 7. The principle is as follows: the oil supply pump 2 opens, the working fluid will be transported to the accumulator 4, when the system pressure exceeds the desired pressure, the safety valve 1 opens, to realize the pressure unloading play a role in protecting the system. With the servo motor 5 driven rotary valve 6 constantly rotating, so that the working fluid alternately into the two piston chamber of the vibration hydraulic cylinder 7, under the action of differential pressure to promote the movement of the piston rod of the hydraulic cylinder, so as to realize the vibration.

### AMESim model of rotary valve control vibration system

**Figure 8:**
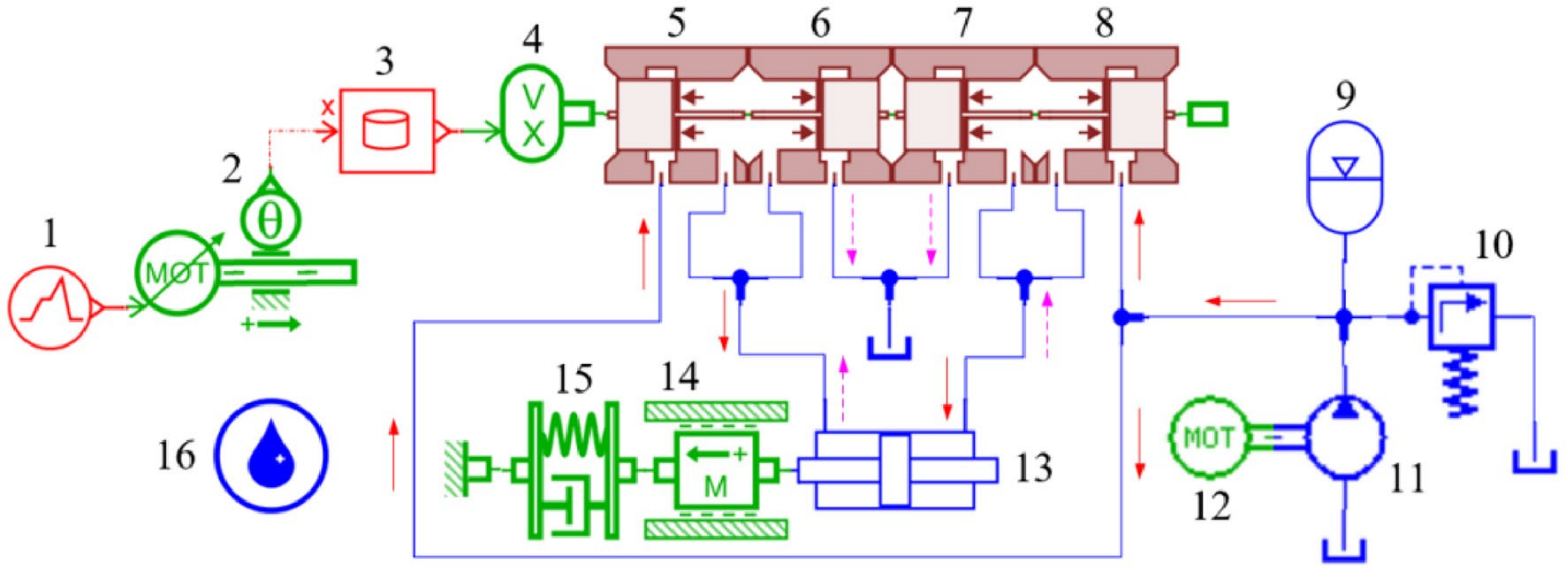
AMESim equivalent model of rotary valve control vibration system. 1. motor speed signal, 2. angle converter, 3 and 4 through-flow area is converted into the axial displacement of the slide valve, 5. 6. 7 and 8 rotary valve structure, 9. accumulator, 10. relief valve, 11 and 12 oil supply pump, 13 and 14 vibration hydraulic cylinder, 15. elastic stiffness and damping, 16. oil property.

According to the structure and occurrence mechanism of the rotary valve control vibration system, the equivalent model of the vibration system is established by using the LMS imagine.Lab AMESim platform, as shown in Fig. 8. The key parameters of the model are shown in Table 3.

**Table 3:** Key parameters.

Project/unit	Numerical value	Project/unit	Numerical value
Rotary valve core radius/mm	50	Motor speed/rpm	500
Vibration hydraulic cylinder piston diameter/mm	99.8	Inlet pressure/MPa	15
Vibration hydraulic cylinder piston rod diameter/mm	55	Accumulator volume/L	6.3
Stroke of vibration hydraulic cylinder/mm	20	Accumulator initial pressure/MPa	2
Equivalent mass of vibration hydraulic cylinder/kg	30	Load spring stiffness/kN m^−1^	100
Oil supply pump displacement/cc·rev^-1^	100	Load damping/kN (m/s)^−1^	10

The zero-opening four-sided slide valve model is used to replace the rotary valve model, which should satisfy the same change rule of the through-flow area of the rotary valve valve port and the slide valve valve port. Define AMESim equivalent model in the slide valve radius and rotary valve valve core radius is the same. Therefore, the maximum displacement *x*_*h*max_ of the slide valve is:

16 $$x_{{hv{\text{max}}}} = \frac{{A_{v\max } }}{2\pi R} = \frac{{x_{v} R\alpha }}{2\pi R} = \frac{{x_{v} R\pi }}{4\pi RZ} = \frac{{x_{v} }}{4Z}$$

where *A*_*v*max_ is the maximum through-flow area of the rotary valve (m^2^).

**Figure 9:**
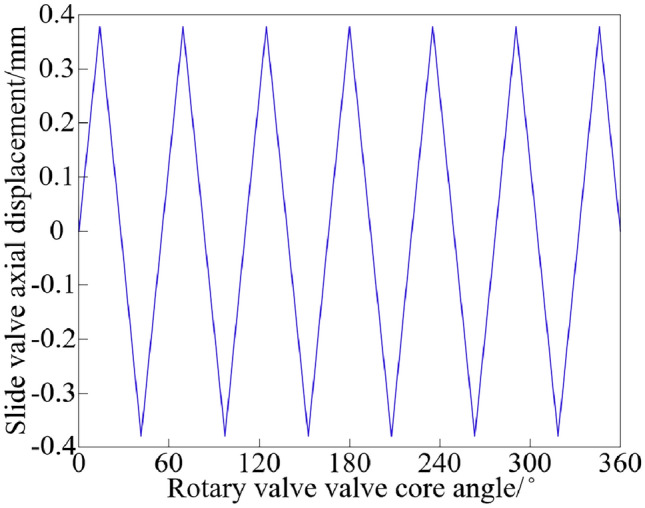
The relationship between the rotary valve valve core angle and the slide valve axial displacement.

Element 3 in Fig. 8 should input the rotary valve valve core angle corresponding to the axial displacement of the slide valve, as shown in Fig. 9.

### Influence of inlet pressures

**Figure 10:**
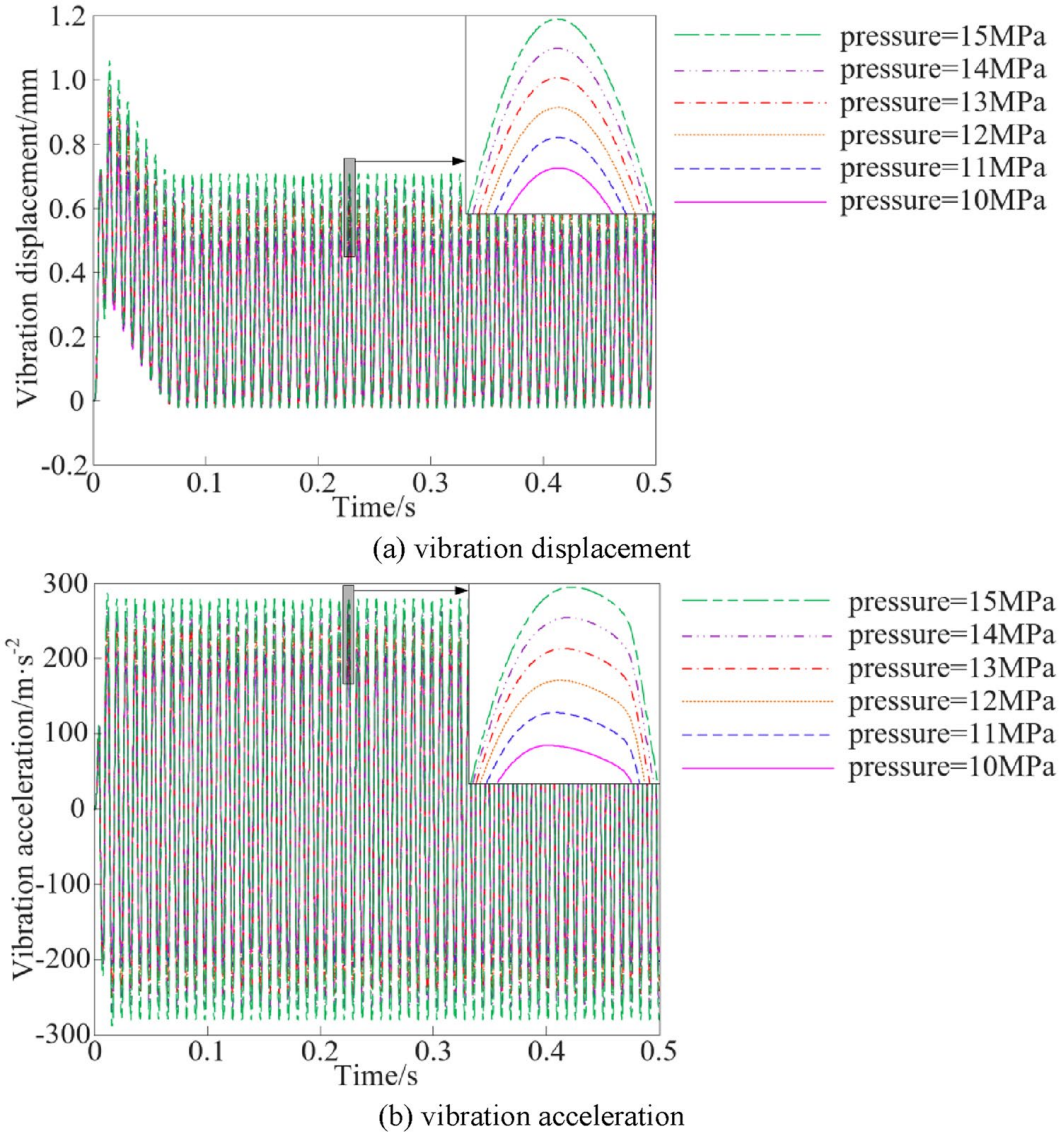
Vibration characteristics at different inlet pressures.

The vibration characteristic curves of the vibration system under different inlet pressure conditions are shown in Fig. 10.

**Figure 11:**
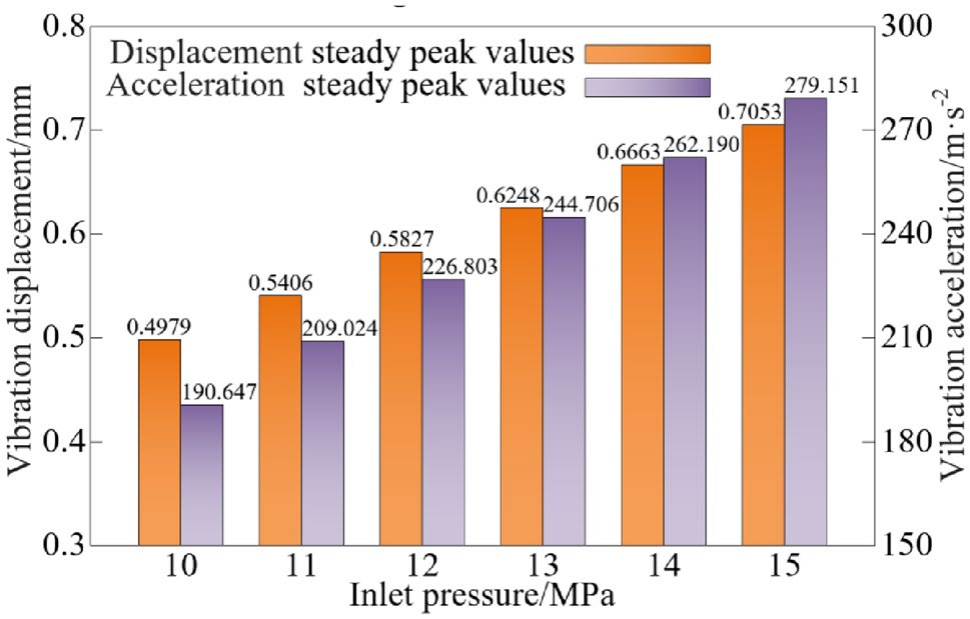
Vibration characteristic steady peak values at different inlet pressures.

The steady peak values statistics of the vibration characteristics of the vibration system when the inlet pressure is different are shown in Fig. 11.

As can be seen from Figs. 10 and 11, when the inlet pressure is increased from 10 to 15 MPa, the steady peak values of vibration displacement and vibration acceleration show an upward trend, and the steady peak values of vibration displacement and vibration acceleration are increased by 41.65% and 46.42%, respectively.

### Influence of motor speed

**Figure 12:**
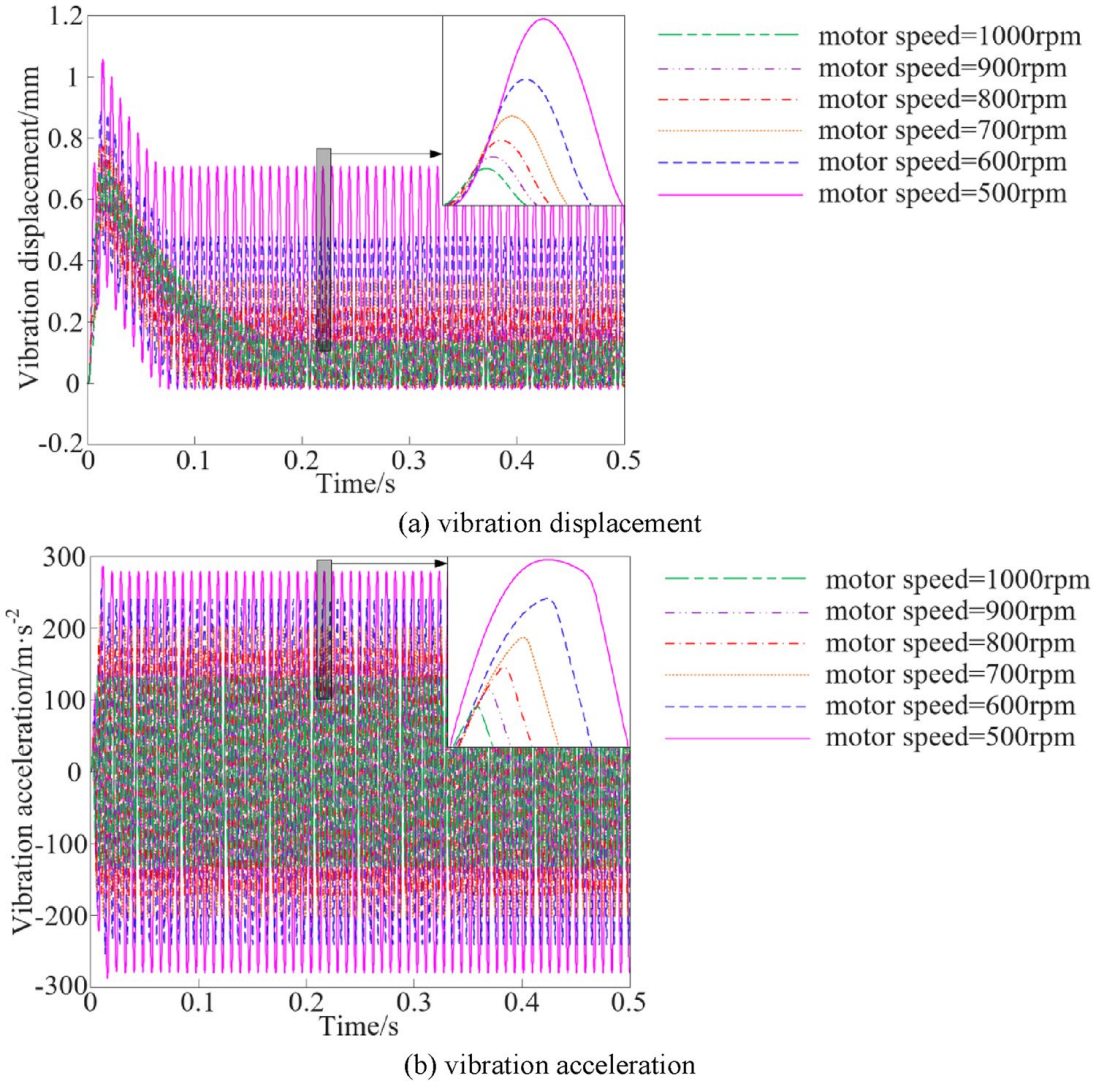
Vibration characteristics at different motor speed.

The vibration characteristic curves of the vibration system under different motor speed conditions are shown in Fig. 12.

**Figure 13:**
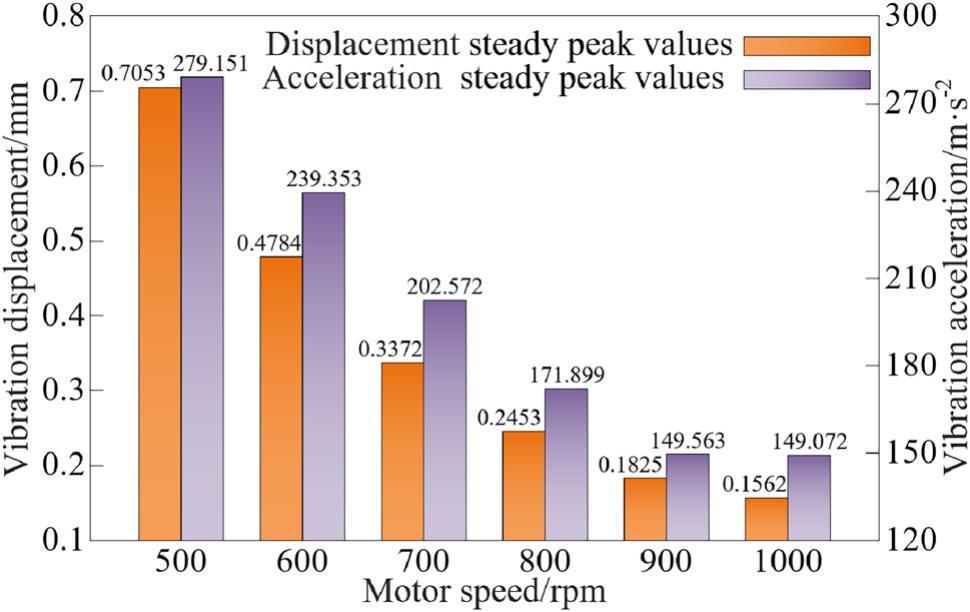
Vibration characteristic steady peak values at different motor speed.

The steady peak values statistics of the vibration characteristics of the vibration system when the motor speed is different are shown in Fig. 13.

As can be seen from Figs. 12 and 13, when the motor speed is increased from 500 to1000 rpm, the steady peak values of vibration displacement and vibration acceleration show an decline trend, and the steady peak values of vibration displacement and vibration acceleration are decline by 77.85% and 46.60%, respectively.

### Influence of oil supply pump displacement

**Figure 14:**
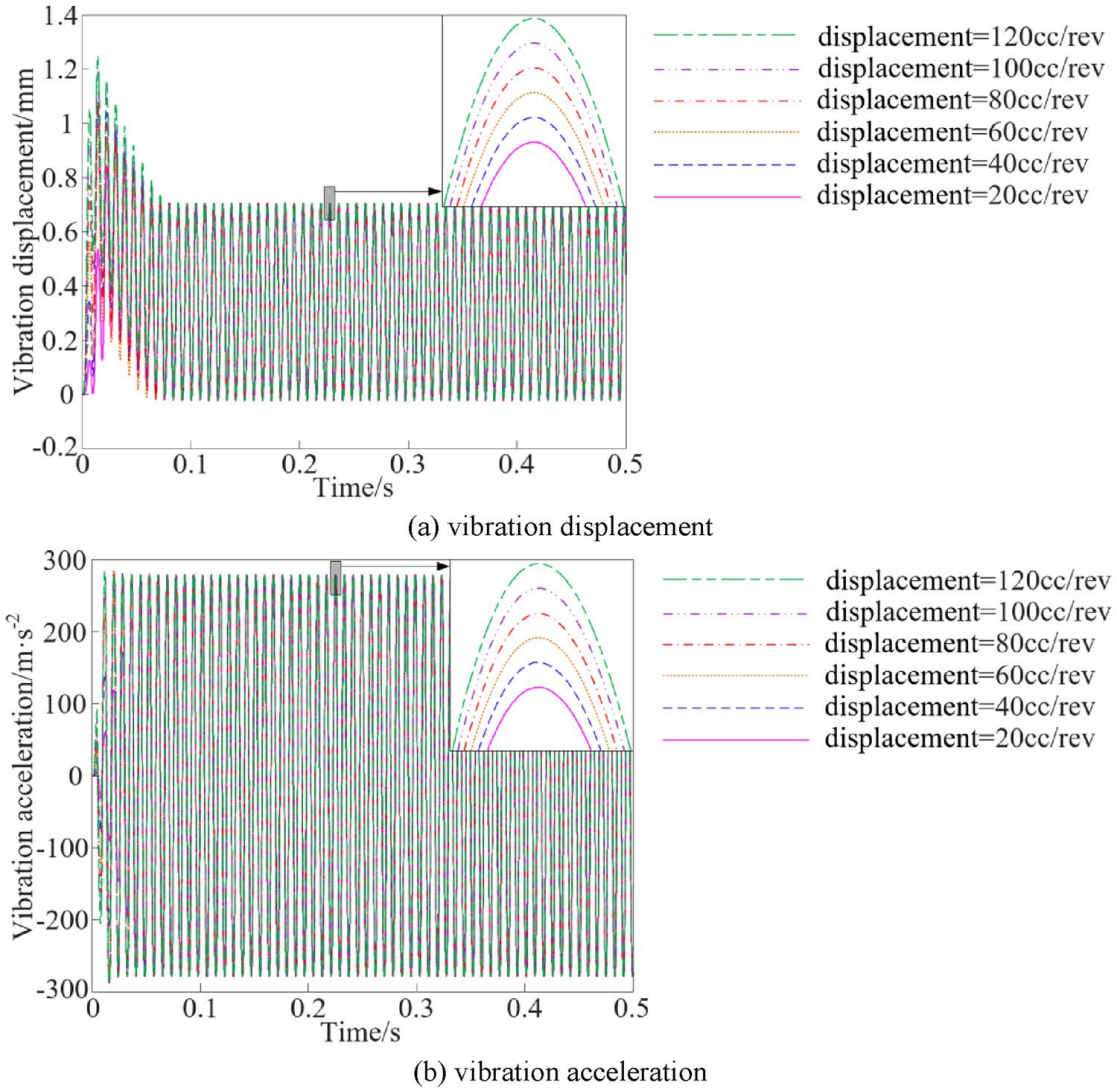
Vibration characteristics at different oil supply pump displacement.

The vibration characteristic curves of the vibration system under different oil supply pump displacement conditions are shown in Fig. 14.

**Figure 15:**
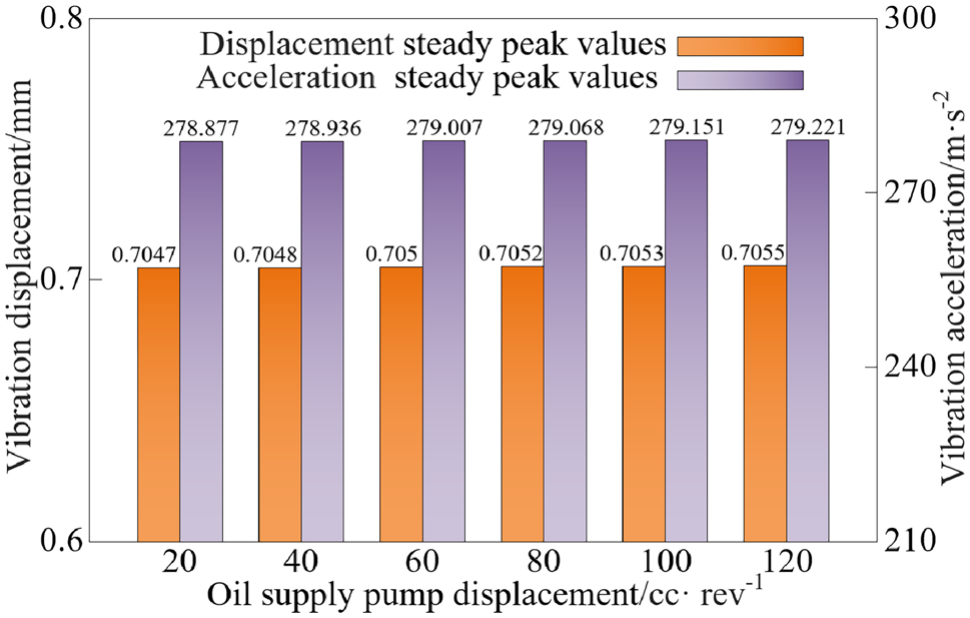
Vibration characteristic steady peak values at different oil supply pump displacement.

The steady peak values statistics of the vibration characteristics of the vibration system when the oil supply pump displacement is different are shown in Fig. 15.

As can be seen from Figs. 14 and 15, when the oil supply pump displacement is increased from 20 to 120 cc/rev, the steady peak values of vibration displacement and vibration acceleration show a slight upward trend, and the steady peak values of vibration displacement and vibration acceleration are increased by 0.11% and 0.12%, respectively, and the effect on the vibration characteristics of the system is not significant.

## Experimental validation

**Figure 16:**
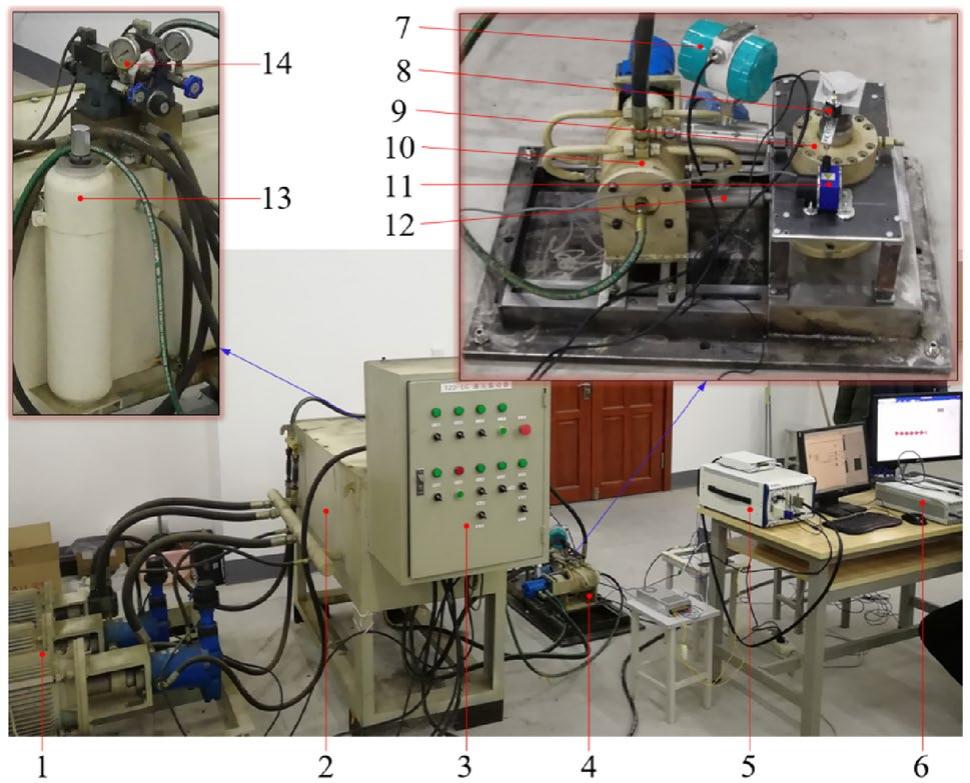
Experiments bench and test site. 1. oil supply pump unit, 2. tank, 3. control cabinet, 4. excitation system, 5. NI acquisition system, 6. DH acquisition system, 7. turbine flowmeter, 8. acceleration sensor, 9. excitation hydraulic cylinder, 10. rotary valve, 11. displacement sensor, 12. pressure transmitter, 13. accumulator, 14. auxiliary valve group.

The rotary valve control vibration system is mainly composed of rotary valve, hydraulic cylinder, oil supply pump, electric control system, other auxiliary hydraulic components and data acquisition system, the experimental bench and the test site is shown in Fig. 16. At the beginning of the experiment, the system is first pre-pressurized by the oil supply pump, then the pressure control system solenoid-directed valve and solenoid relief valve are used to adjust the initial pressure of the hydraulic cylinder and the accumulator pressure to reach the preset value, keeping the experimental and simulation conditions consistent, and finally the motor is turned on for the experiment. To ensure the reliability of the test results, three experiments were conducted under the same conditions, and the average value was taken as the sample data for analyzing the experiment results. Vibration system experiment bench equipment model and parameters are listed in Table 4.

**Table 4:** Experimental bench equipment models and parameters.

Project/unit	Model	Numerical value
Oil supply pump/L min^−1^	25DCY14	37.5
Pressure transmitter/MPa	BP800	0–40
Motor speed/rpm	LCMT15M	0–1500
Accumulator/L	NXQAB40/31.5-F	6.3L
Electromagnetic relief valve/MPa	DBW10B-1	0–31.5
Electromagnetic reversing valve/MPa	4WE10J31/CG24N9Z5	0–31.5

### Experimental verification of rotary valve output characteristics

**Figure 17:**
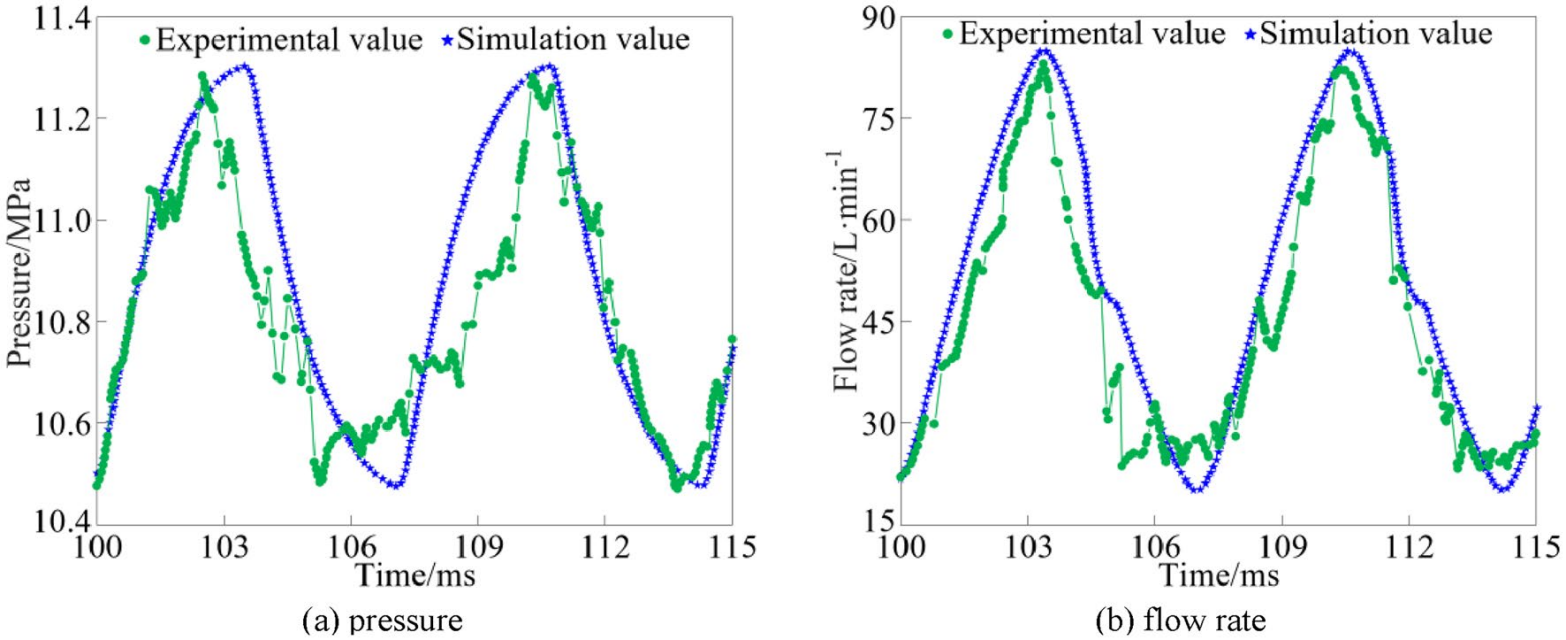
Rotary valve output characteristics verification.

The comparison of simulated and experimental results of the output characteristics of the rotary valve at inlet pressure of 15 MPa and motor speed of 500 rpm is shown in Fig. 17.

As can be seen from Fig. 17, the simulation and experimental results of the output pressure peak of the rotary valve are 11.34 MPa and 11.28 MPa, respectively, and the simulation and experimental results of the output flow rate peak are 84.42L/min and 82.73L/min, respectively. The error between the experimental results of the output pressure and the simulation results is 0.532%, and the error between the experimental results of the output flow rate and the simulation results is 2.043%, and the experimental results are in high agreement with the simulation results.

### Experimental verification of system vibration characteristics

**Figure 18:**
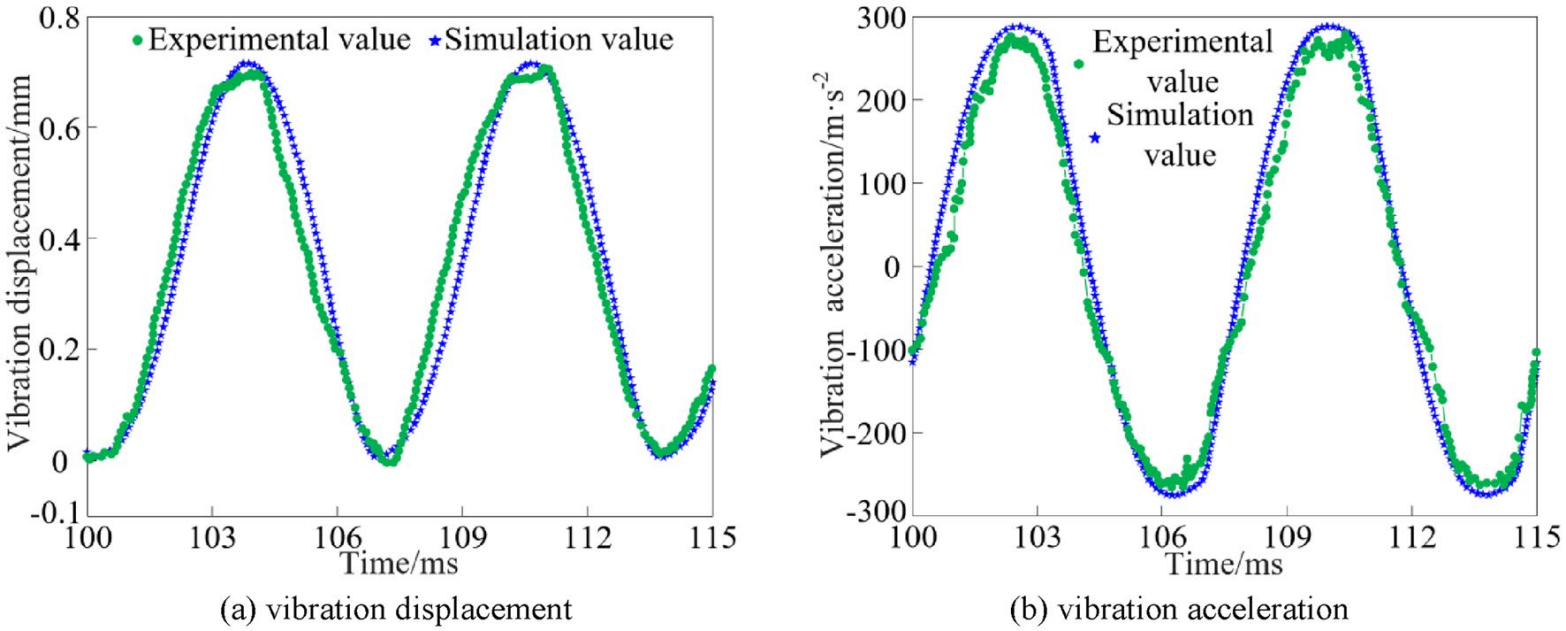
Verification of vibration characteristics.

The comparison of the simulated and experimental results of the vibration characteristics of the system at an inlet pressure of 15 MPa is shown in Fig. 18.

As can be seen from Fig. 18, the simulation and experimental results of the steady peak value of vibration displacement of the system are 0.7053 mm and 0.6905 mm, respectively, and the simulation and experimental results of the steady peak value of vibration acceleration of the system are 279.151 m/s^2^ and 268.624 m/s^2^, respectively. The error between the experimental results of the vibration displacement steady peak value and the simulation results is 2.143%, and the error between the experimental results and the simulation results is 3.919%, and the experimental results are highly consistent with the simulation results.

In summary, it can be seen that the system vibration characteristic curve waveform is approximately sinusoidal, and the waveform conforms to the design principle of rotary valve. Neglecting the error caused by environmental factors during the experiment, it can be considered that the simulation results and the experimental results are highly consistent, proving the rationality of the rotary valve structure and the correctness of the vibration system. The experimental results of the vibration characteristics of the steady peak value of a small amplitude deviation and fluctuations, indicating that the rotary valve flow distribution process of pressure loss is small.

## Conclusion

A study proposes a rotary valve control vibration system and builds an experimental bench for verification. The method of MRF is used to analyze the change rule in output characteristics of the rotary valve. An AMESim model is developed for the rotary valve control vibration system to investigate the influence of multiple parameters on the system’s vibration characteristics. The following conclusions are drawn:

1. The through-flow area of the rotary valve has a direct impact on the output pressure and flow rate. The trend of change in the through-flow area corresponds to the trend of change in the output pressure and flow rate.
2. The steady peak value of the system’s vibration characteristic increases with higher inlet pressure, while it decreases with an increase in motor speed. The influence of oil supply pump displacement on the vibration characteristics of the system is not significant.
3. The experimental and simulation results for the output pressure and flow rate of the rotary valve exhibit errors of 0.532% and 2.043%, respectively. The errors between the experimental and simulation results for the steady peak value of vibration displacement and acceleration was found to be 2.143% and 3.919%, respectively.
4. The waveform of the system vibration characteristic curve exhibits a sinusoidal pattern, and the experimental waveform aligns well with the simulated waveform. The experimental results demonstrate smaller deviations and fluctuations in the stable peak values of the vibration characteristics compared to the simulation results, indicating a reduced pressure loss during the flow distribution process of the rotary valve. To enhance the system’s vibration characteristics, it is recommended to increase the inlet pressure and appropriately decrease the motor speed.

## Data Availability

The datasets used and/or analysed during the current study available from the corresponding author on reasonable request.
